# Evaluation of early implementations of antibiotic stewardship program initiatives in nine Dutch hospitals

**DOI:** 10.1186/2047-2994-3-33

**Published:** 2014-10-23

**Authors:** Maarten van Limburg, Bhanu Sinha, Jerome R Lo-Ten-Foe, Julia EWC van Gemert-Pijnen

**Affiliations:** Department Psychology Health and Technology, Faculty Behavioral, Management and Social Sciences, University of Twente, Enschede, the Netherlands; Department of Medical Microbiology, University of Groningen, University Medical Center Groningen, Groningen, the Netherlands

**Keywords:** Evaluation, Implementation, Antibiotic stewardship programs, Maturity, Hospital infections

## Abstract

**Background:**

Antibiotic resistance is a global threat to patient safety and care. In response, hospitals start antibiotic stewardship programs to optimise antibiotic use. Expert-based guidelines recommend strategies to implement such programs, but local implementations may differ per hospital. Earlier published assessments determine maturity of antibiotic stewardship programs based on expert-based structure indicators, but they disregard that there may be valid deviations from these expert-based programs.

**Aim:**

To analyse the progress and barriers of local implementations of antibiotic stewardship programs with stakeholders in nine Dutch hospitals and to develop a toolkit that guides implementing local antibiotic stewardship programs.

**Methods:**

An online questionnaire based on published guidelines and recommendations, conducted with seven clinical microbiologists, seven infectious disease physicians and five clinical pharmacists at nine Dutch hospitals.

**Results:**

Results show local differences in antibiotic stewardship programs and the uptake of interventions in hospitals. Antibiotic guidelines and antibiotic teams are the most extensively implemented interventions. Education, decision support and audit-feedback are deemed important interventions and they are either piloted in implementations at academic hospitals or in preparation for application in non-academic hospitals. Other interventions that are recommended in guidelines - benchmarking, restriction and antibiotic formulary - appear to have a lower priority. Automatic stop-order, pre-authorization, automatic substitution, antibiotic cycling are not deemed to be worthwhile according to respondents.

**Conclusion:**

There are extensive local differences in the implementation of antibiotic stewardship interventions. These differences suggest a need to further explore the rationale behind the choice of interventions in antibiotic stewardship programs. Rather than reporting this rationale, this study reports where rationale can play a key role in the implementation of antibiotic stewardship programs. A one-size-fits-all solution is unfeasible as there may be barriers or valid reasons for local experts to deviate from expert-based guidelines. Local experts can be supported with a toolkit containing advice based on possible barriers and considerations. These parameters can be used to customise an implementation of antibiotic stewardship programs to local needs (while retaining its expert-based foundation).

**Electronic supplementary material:**

The online version of this article (doi:10.1186/2047-2994-3-33) contains supplementary material, which is available to authorized users.

## Background

Antibiotic resistance is an increasing worldwide threat to patient safety and quality of care [1, 2]. A correlation between antibiotic (over) use and increasing antibiotic resistance is widely acknowledged [3]. Hospitals can influence antibiotic use with improved, prudent antibiotic prescription, as up to 50% of prescribed antibiotics may be inappropriate or even unnecessary [4]. To curb the increasing resistance, hospitals started antibiotic stewardship programs (ASPs) as quality initiatives for infection prevention and control. An ASP ensures proper use of antibiotics with the best patient outcomes, less risk of adverse effects, optimal cost-effectiveness and to reduce or stabilise levels of resistance [5]. Guidelines from professional societies, such as the Infectious Diseases Society of America/Society for Healthcare Epidemiology of America (IDSA/SHEA) recommend expert-based strategies and interventions to implement ASPs in hospitals [6–8]. In the Netherlands, the Dutch Working Party on Antibiotic Policy (SWAB) is responsible for guideline development, education and surveillance for optimal antibiotic use. This organisation recently published a vision document, stressing the need for Dutch hospitals to form an ASP team and start planning future ASP initiatives as per January 2014 [9].

There is no consistent approach in local implementations of ASP in hospitals [10]. Hospitals combine expert-based strategies and subsequent ASP interventions in many different variations [4]. In a prior systematic review, neither a consistent implementation strategy nor a sufficiently described implementation rationale could be identified in international ASP studies (van Limburg M, Köck R, Karreman J, Sinha B, de Jong N, Wentzel J, Friedrich A, Hendrix R, van Gemert-Pijnen J, “Towards an Implementation Strategy for Antibiotic/Antimicrobial Stewardship: A Systematic Review”, Under review). To understand how these local implementations of ASP take form it is important to use a bottom-up analysis from within hospitals. In this approach, we involve the relevant local stakeholders in the hospitals we aim to assist with implementing ASP, as opposed to an expert-driven or authoritative top-down approach.

Progression in implementation can be assessed with the concept of ‘maturity’. Maturity models were first coined by Paulk et al [11] and are used in many disciplines to model or evaluate improvement of organisations and processes [12]. Maturity models describe a levelled progress of how, in this case, an ASP implementation gradually improves from an ad-hoc state to a structured and managed state and eventually to a measurable and self-optimizing state [11].

Maturity assessments are already used in the context of ASP. Earlier maturity assessments of ASPs were done by the Antibiotic Strategy International group who introduced a comprehensive list of potential structure indicators to assess maturity of ASP in hospitals in several European countries [13]. In short, this means that maturity of ASP depends on the number of expert-based structure indicators that are implemented as part of ASP in a hospital.

This study does not attempt to assess maturity by scoring a completeness of an ideal program, but analyses differences in local implementations of ASP interventions that are recommended in guidelines. Our aim is not primarily to identify which interventions are or should be implemented, but more importantly, to identify where rationale plays a role in implementing ASP interventions and what the consequences are for expert-based ASP strategies. The present study is the first step in our bottom-up assessment with local ASP experts to give an overview of which recommended interventions are implemented, or are being developed, combined with an importance analysis of these interventions. Incongruent responses from stakeholders show where possible difficulties or barriers surface when implementing ASP conform currently available expert guidelines and indicate possibilities for decision support. A follow-up study is required to understand the deeper rationale behind these differences and barriers and how local experts can be assisted with relevant implementation advice.

## Methods

An online questionnaire using LimeSurvey was made available from August to November 2013. Clinical microbiologists, clinical pharmacists and infectious disease physicians were approached as these expert types are identified as being key-stakeholders in ASP (van Limburg M, Köck R, Karreman J, Sinha B, de Jong N, Wentzel J, Friedrich A, Hendrix R, van Gemert-Pijnen J, “Towards an Implementation Strategy for Antibiotic/Antimicrobial Stewardship: A Systematic Review”, Under review). Ethical approval was given after revision by an internal representative of the ethical board (reference: BCE14039) and no further ethical review procedures were deemed necessary. We sent a questionnaire invitation to known infection control experts recruited in earlier research projects. These experts represented academic and non-academic hospitals in the Dutch eastern border region and they were asked to distribute the questionnaire among clinical microbiologists, clinical pharmacists and infectious disease physicians tasked with ASP in their hospitals. As at the time of the questionnaire ASP teams were not necessarily formed at the hospitals yet, we chose to approach key stakeholders rather than teams directly. We also analysed the response of key stakeholders individually (not clustered per hospital) as stakeholders may have possible differences in views on ASP interventions (even within hospitals).

The questionnaire was structured around ten implementation topics based on a literature review (see Table 1). The topics that were addressed involved ASP program initiatives, presence and importance of commonly used interventions and their possible characteristics, and possible outcomes and effects relevant for assessing evidence for the effectiveness of ASP. The extraction of implementation topics concerning ASP maturity or implementation assessments, attitude or opinion surveys towards ASP or practical implementation toolkits for ASP are presented in detail in Table 1.

**Table 1:** **Overview of literature scan and extracted implementation topics**

#	Author (s)	Year	Title	Study description	Extracted implementation topics
**1**	Bannan, Buono, McLaws, Gottlieb	2009	Survey of medical staff attitudes to an antibiotic approval and stewardship program	Design:	- restriction as intervention
				Questionnaire with 40 questions focused on restriction and approval	- authorization as intervention
				Interest:	- advice as communication
					- education as intervention
				Attitude	- stop-order (withholding pharmacy) as intervention
					- costs, appropriate use, resistance, time as outcomes
					- pager as communication
					- possible stakeholders in team
**2**	Barlam, DiVall	2006	Antibiotic-stewardship practices at top academic centers throughout the united states and at hospitals throughout Massachusetts	Design:	- multifaceted programs
				Two surveys	- time of start with ASP
				Interest:	- funding/financial support
				ASP components	- (formulary) restriction as intervention
					- solicited input from ID as communication
					- costs, improved use, adverse effects, resistance, compliance, DDDs, clinical outcomes as outcomes
					- aiming prophylaxis
					- aiming only targeted antibiotics
					- aiming antibiotic therapy at order
					- aiming initial therapy
					- recommendations as intervention (day 3 bundle)
					- culture data as communication
					- possible stakeholders in team
					- approval as intervention
					- review as communication
					- consult as communication
					- computerized order entry as communication
					- stop-order as intervention
					- IV-PO switch as intervention
					- clinical practical guidelines as intervention
					- evaluation as intervention (benchmarking)
					- support and time needed from physicians
					- rounds, didactics, program, consults/feedback as education
3*	Burgmann, Janata, Allerberger, Frank	2008	Hospital antibiotic management in Austria – results of the ABS maturity survey of the ABS International group	Design:	- data evaluation as intervention (benchmarking)
				Survey	- AB consumption data as outcomes
				Interest:	- hospital/department/ward levels of benchmarking
				5 categories of maturity	- feedback of benchmarking as communication
					- possible stakeholders in team
					- guidelines for dosage, drug costs, IV-PO switch
					- guidelines for antibiotic treatment
					- guidelines for prophylaxis
					- education as intervention (seminars, literature)
					- financial resources
					- cooperation with other hospitals
**4**	Buyle, Metz-Gercek, Mechtler, Kern, Robays, Vogelaers, Struelens	2013	Development and validation of potential structure indicators for evaluating antimicrobial stewardship programmes in European hospitals	Design:	- bedside advice as communication
				Expert panel + validation survey	- rounds as intervention
					- frequency of team meetings
				Interest:	- audit as intervention
				Potential structure indicators for ASP	- possible stakeholders in team
					- formulary as intervention
					- updating formulary
					- stop order as intervention
					- guidelines for microbiological documented therapy, empirical therapy, prophylaxis, iv-po switches
					- updating guidelines
					- clinical decision aid as IT
					- mandate from management
					- FTEs
					- Education as interventions
					- passive methods, interactive methods as education
					- evaluation as intervention
					- resistance data, consumption data,
					- hospital/department/ward levels of benchmarking
					- total DDDs, # of infections as outcomes
**5**	Cooke, Alexander, Charani, Hand, Hills, Howard, Jamieson, Lawson, Richardson, Wade	2010	Antimicrobial stewardship: an evidence-based, antimicrobial self-assessment toolkit (ASAT) for acute hospitals	Design:	- guidelines as intervention
				ASAT toolkit (checklist)	- formulary as intervention
				Interest:	- restriction as intervention
				Levels of antimicrobial stewardship	- IV-PO switches as intervention
					- guidelines for prophylaxis as intervention
					- adherence as outcome
					- education as intervention
					- training as education
					- information systems as IT
					- digital prescribing as IT
					- possible stakeholders in team
**6**	Dumartin, Rogues, Amadeo, Pefau, Venier, Parneix, Maurain	2011	Antibiotic stewardship programmes: legal framework and structure and process indicator in Southwestern French hospitals, 2005–2008	Design:	- frequency in meetings
				Survey	- available human resources
				Interest:	- digital prescription, pharmaceutical analysis, dispensation, digital link between lab, pharm, wards as IT
				Checking whether legal framework is present	- restriction as intervention
					- stop order as intervention
					- first-line, prophylaxis as guidelines
					- audits as intervention/communication
					- evaluation feedback as communication
					- education as intervention
					- Formulary as intervention
					- ab consumption as benchmarking
					- DDDs, resistance as outcomes (and communication)
					- possible stakeholders in team
**7**	van Gastel, Costers, Peetermans, Struelens	2010	Nationwide implementation of antibiotic management teams in Belgian hospitals: a self-reporting survey	Design:	- Possible stakeholders in team
				Questionnaire	- consultation per phone, email, intranet, face-to-face, staff meetings as communication
				Interest:	- formulary as intervention
				Level of AMT activities	- guidelines for empirical and prophylaxis
					- updates of formulary and guidelines
					- restriction as intervention
					- approval/review as intervention
					- concurrent review/audit as intervention
					- de-escalation as intervention
					- stop order as intervention
					- order forms as intervention
					- IV-PO switch as intervention
					- consumption and resistance as outcomes
					- by hospital/unit or by antibiotic type
					- feedback of outcomes
**8**	Greater New York Hospital Association	2011	Antimicrobial stewardship toolkit	Design:	- benchmark and review antibiotic use (patterns)
				Best practice	- review resistance
				Interest:	- IT infrastructure
				Implementation toolkit	- possible stakeholders in team
					- aim for common infections, pathogens, agents
					- rollout: hospital vs. ward
					- available resources
					- strategy:
					- guidelines for diagnosis, treatment, duration, dose optimization, IV-PO, streamlining/de-escalation
					- formulary as intervention
					- restriction as intervention
					- education as intervention
					- prospective review as intervention
					- stickers, notes, face-to-face as communication
					- data collection (benchmarking)
					- usage, clinical, microbiologic, costs as data
**9**	Hulscher, Grol, van der Meer	2010	Antibiotic prescribing in hospitals: a social and behavioral scientific approach	Design:	- formulary as intervention
				Review	- order form as intervention
				Interest:	- restriction as intervention
				socio-cultural factors of ASP	- stop orders as intervention
					- infection control committee
					- guidelines as intervention
					- review as intervention
					- rounds as intervention
					- telephone advice as intervention
					- improve infrastructure
					- education as intervention
					- conferences, seminars, skill training programs as education
					- individual instructions (outreach, academic detailing)
					- feedback of outcomes
					- decision support via IT
**10**	Nault, Beaudoin, Thirion, Gosselin, Cossette, Valiquette	2008	Antimicrobial stewardship in acute care centres: survey of 68 hospitals in Quebec	Design:	- Duration of ASP or busy setting up
				Questionnaire	- distributed units, DDDs, acquisition costs as benchmarking data
				Interest:	- direct interaction as intervention (written or phone)
				Proportion and nature of programs	- education as intervention
					- stop orders as intervention
					- auto substitution
					- formulary restriction as interventions
					- local guidelines as intervention
					- preauthorization as intervention
					- antibiotic cycling as intervention
					- decision support systems as intervention
					- possible stakeholders in team
**11**	Pulcini, Williams, Molinari, Davey, Nathwani	2011	Junior doctors’ knowledge and perceptions of antibiotic resistance and prescribing: a survey in France and Scotland	Design:	- local guidelines as intervention
				Survey	- presence of team-
				Interest:	- approval as intervention
				Perception and prescribing practice	- IV-PO switch protocol
					- advice from ID physician, senior, microbiologist, pharmacist or team as intervention
					- face-to-face, phone, consult upon request as communication
					- lectures, workshops, informal education, web-based learning, self-directed learning as education
					- possible stakeholders in team
					- computer aided prescribing as IT
					- resistance data availability
**12**	Thern	2013	Selection of hospital antimicrobial prescribing quality indicators:	Design:	- possible stakeholders in team
			a consensus among German antibiotic stewardship (ABS) networkers	Review+	- frequency of meetings
				questionnaire	- mandate
				Interest:	- drug use, resistance rates as data
				Indicators for quality of AB prescribing	- formulary as intervention
					- updating formulary
					- restriction/approval as intervention
					- guidelines for empiric therapy, IV-PO, dosing, prophylaxis,
					- rounds as intervention
					- education as intervention
					- guidance or assisted decision analysis via IT
**13**	Trivedi, Rosenberg	2013	The state of antimicrobial stewardship programs in California	Design:	- implemented or planned ASP
				Survey	- time of start with ASP
				Interest:	- possible stakeholders in team
				State of ASP	- FTE availability
					- - funding
					- benchmarking as intervention
					- DDDs, DOTs, costs, acceptance of recommendations, improved susceptibility patterns as data
					- use of IT in ASP
					- electronic health record, digital prescription, electronic medication administration records as IT
					- formulary restriction as intervention
					- ID physician consult as intervention
					- audit as intervention
					- prior approval as intervention
					- auto stop orders as intervention
					- verbal approval as intervention
					- pre-authorization as intervention
					- education as intervention
					- guidelines as intervention
					- IV-PO switch as intervention
					- streamlining/de-escalation as intervention
					- order forms as intervention

*) DDDs: daily defined doses; DOT: days of therapy; FTE: full-time equivalent; ID: infectious diseases; IV-PO: intravenous-per os; IT: information technology.

The final questionnaire was adapted for the Dutch healthcare context, reduced and validated by microbiologists to have a final scope of 47 questions for an acceptable answering duration of 15–20 minutes. A literal English translation of the Dutch questionnaire can be found in Additional file 1 for reference.

Responses were imported into SPSS21 for statistical analysis. Incomplete responses were excluded. Frequency cross-tables of responses stratified by hospital type (academic/non-academic) were used for reporting all results, as we anticipated differences between the two hospital types [10].

## Results

### Response

A total of 27 responses were collected. Eight respondents started answering the first few questions but did not complete the questionnaire and had to be excluded from the results. Based on the first questions we could identify that these were three clinical microbiologists, one infectious disease physician and four clinical pharmacists. Nineteen respondents completed the questionnaire, and the final sample consists of seven clinical microbiologists, seven infectious disease physicians and five clinical pharmacists from three academic hospitals and six non-academic (general tertiary care) hospitals.

### Antibiotic stewardship program initiatives

The three included academic hospitals have implemented an ASP. At the non-academic hospitals, ASP initiatives are in development at five hospitals while one hospital has implemented an ASP. Almost all ASP initiatives were started from 2012 onwards.

Respondents from academic hospitals seemed more satisfied with current mandate from hospital management, available budgets and assigned full-time equivalents (FTEs) than their non-academic peers. No consensus was found in their opinions regarding satisfaction with the level of formalisation of ASP with, for example, documentation or task descriptions.

Academic hospitals targeted multiple wards in their ASP implementations, but the type of wards varied. Generally these were all wards with high risk of infections and with relatively high antibiotic use. Most non-academic hospitals did not yet implement ASP. There, however, is a strong consensus that eventually ASPs should be implemented throughout the entire hospital.

### Implementation of ASP interventions

Table 2 provides an overview of the maturity assessment as to whether common ASP interventions were already implemented, in development, considered necessary or considered unnecessary. The following paragraphs discuss the interventions in more detail.

**Table 2:** **Maturity of ASP interventions in academic and non-academic hospitals**

	Academic hospitals					Non-academic hospitals				
	Impl	In dev	Need	Unneed	N/A*	Impl	In dev	Need	Unneed	N/A*
Antibiotic team	6 (75%)	2 (25%)		-	-	6 (55%)	5 (45%)	-	-	-
(Local) antibiotic guidelines	7 (88%)	1 (12%)	-	-	-	9 (82%)	2 (18%)	-	-	-
Antibiotic formulary	7 (88%)	1 (13%)	-	-	-	8 (73%)	2 (18%)	1 (9%)	-	-
Audit-and-feedback	3 (38%)	2 (25%)	2 (25%)	-	1 (13%)	-	4 (36%)	6 (55%)	1 (9%)	-
Education	4 (50%)	4 (50%)	-	-	-	2 (18%)	8 (73%)	1 (9%)	-	-
Information systems for ASP	2 (25%)	1 (13%)	5 (63%)	-	-	2 (18%)	4 (36%)	3 (27%)	-	2 (18%)
Benchmarking	4 (50%)	3 (38%)	-	1 (13%)	-	1 (9%)	7 (64%)	2 (18%)	-	1 (9%)
Restriction	2 (25%)	4 (50%)	1 (13%)	1 (13%)			5 (45%)	3 (27%)	1 (9%)	2 (18%)
Academic detailing	1 (13%)	3 (38%)	3 (38%)	-	1 (13%)	2 (18%)	2 (18%)	4 (36%)	-	3 (27%)
Automatic stop-order	-	1 (13%)	4 (50%)	2 (25%)	1 (13%)	-	4 (36%)	3 (27%)	2 (18%)	2 (18%)
Pre-authorization	1 (13%)	1 (13%)	3 (38%)	1 (13%)	2 (25%)	-	2 (18%)	3 (27%)	1 (9%)	5 (45%)
Automatic substitution	-	1 (13%)	2 (25%)	5 (63%)		-	1 (9%)	3 (27%)	2 (18%)	5 (45%)
Antibiotic cycling	-	1 (13%)	1 (13%)	4 (50%)	2 (25%)	-	-	5 (45%)	2 (18%)	4 (36%)

*) Impl: implemented; in dev: in development; need: are needed; unneed: are unneeded; N/A: no answer or not applicable.

### Antibiotic team

In the early stages of ASP implementation, an antibiotic team (usually called A-team for obvious reasons) is mostly responsible for preparing an ASP. Subsequently, their tasks change to monitoring ASP effectiveness and performing important tasks that are part of the program. Based on the response, we can assume antibiotic teams were either already formed or in preparation.

Table 3 shows the composition of these teams. In both types of hospitals the antibiotic teams consist of at least infectious disease physicians, clinical microbiologists and clinical pharmacists. These are also the stakeholders recommended to be at least present according to the national guidelines endorsed by SWAB [9]. Other stakeholders are involved in different team configurations but there is no consensus on which of these other stakeholders should be definite members of the antibiotic team. In both types of hospitals it is clear that management and attending physicians have no active role in the team.

**Table 3:** **Composition of antibiotic team in academic and non-academic hospitals**

	Academic hospitals					Non-academic hospitals				
	Impl	In dev	Need	Unneed	N/A*	Impl	In dev	Need	Unneed	N/A*
Clinical microbiologist	8 (100%)	X		-	-	9 (82%)	X	1 (9%)	-	1 (9%)
Infectious disease physician	8 (100%)	X		-	-	5 (45%)	X	-	1 (9%)	5 (45%)
Clinical pharmacist	7 (88%)	X		1 (13%)	-	9 (82%)	X	1 (9%)	-	1 (9%)
Member of antibiotic committee	5 (63%)	X	1 (13%)	-	2 (25%)	4 (36%)	X	3 (27%)	1 (9%)	3 (27%)
Prescribing physician	4 (50%)	X	-	3 (38%)	1 (13%)	-	X	4 (36%)	3 (27%)	4 (36%)
Hygienist	1 (13%)	X	3 (38%)	3 (38%)	1 (13%)	1 (9%)	X	3 (27%)	4 (36%)	3 (27%)
IT specialist	-	X	3 (38%)	4 (50%)	1 (13%)	-	X	4 (36%)	5 (45%)	2 (18%)
Nurse	-	X	3 (38%)	3 (38%)	2 (25%)	-	X	2 (18%)	5 (45%)	4 (36%)
Epidemiologist	1 (13%)	X	4 (50%)	1 (13%)	2 (25%)	-	X	1 (9%)	4 (36%)	6 (55%)
Management	-	X	-	6 (75%)	1 (13%)	-	X	2 (18%)	6 (55%)	2 (18%)
Supervising physician	1 (13%)	X	1 (13%)	5 (63%)	1 (13%)	-	X	1 (9%)	6 (55%)	4 (36%)

*) Impl: implemented; in dev: in development; need: are needed; unneed: are unneeded; N/A: no answer or not applicable.

In non-academic hospitals, the frequency of infectious disease physicians in the teams is lower compared to academic hospitals. This can be explained by the probability that non-academic hospitals have no or too few (e.g. shared with other local hospitals) infectious disease physicians available, hence they cannot take part in their antibiotic team.

### (Local) Antibiotic guidelines

There are national antibiotic guidelines to which Dutch hospitals have to comply, based on evidence or expert-based guidelines and policies established in academic hospitals (e.g. available at http://www.swabid.nl). Hospitals are at liberty to use these national guidelines in full or adapt them slightly into local antibiotic guidelines.

Table 4 shows that academic hospitals have most common types of antibiotic guidelines locally implemented and overall, respondents were satisfied with currently available antibiotic guidelines in their hospitals. They responded that guidelines for intravenous-per os switches and for de-escalation are not yet available, but needed.

**Table 4:** **Status of antibiotic guidelines in academic and non-academic hospitals**

	Academic hospitals					Non-academic hospitals				
	Impl	In dev	Need	Unneed	N/A*	Impl	In dev	Need	Unneed	N/A*
Diagnosis of infections	8 (100%)	-	-	-	-	4 (36%)	-	3 (27%)	-	4 (36%)
Treatment of infections	8 (100%)	-	-	-	-	7 (64%)	2 (18%)	-	-	2 (18%)
Antibiotic therapy	8 (100%)	-	-	-	-	10 (91%)	1 (9%)	-	-	-
Duration of therapy	7 (88%)	-	1 (13%)	-	-	5 (45%)	2 (18%)	3 (27%)	-	-
Prophylaxis	8 (100%)	-	-	-	-	10 (91%)	1 (9%)	-	-	-
IV-PO switches	5 (63%)	-	3 (38%)	-	-	3 (27%)	4 (36%)	4 (36%)	-	-
De-escalation/streamlining	3 (38%)	1 (13%)	4 (50%)	-	-	1 (9%)	4 (36%)	5 (45%)	-	-

*) Impl: implemented; in dev: in development; need: are needed; unneed: are unneeded; N/A: no answer or not applicable.

The situation in non-academic hospitals is different. Guidelines for treatment of infections, antibiotic therapy and prophylaxis are implemented (these are all available as national antibiotic guidelines). However, other antibiotic guidelines are incomplete and not as available when compared to academic hospitals. Respondents from non-academic hospitals agree that additional antibiotic guidelines are needed. It is interesting to see that exactly those guidelines that academic hospitals expressed as being needed (intravenous-per os switches and de-escalation) are in development at non-academic hospitals.

### Antibiotic formulary

An antibiotic formulary contains an overview of indications and favourable antibiotic treatment (s). In the Netherlands, these formularies are usually introduced and maintained by the clinical microbiologists. Overall, an antibiotic formulary is present in both types of hospitals.

### Audit-and-feedback

Audits are interactions with prescribers to influence the way they prescribe for antibiotic therapy. These audits can be prospective, thus directly influence therapy with feedback at patient level, or retrospective where the prescribed antibiotics are assessed and reported back to prescribers.

**Figure 1:**
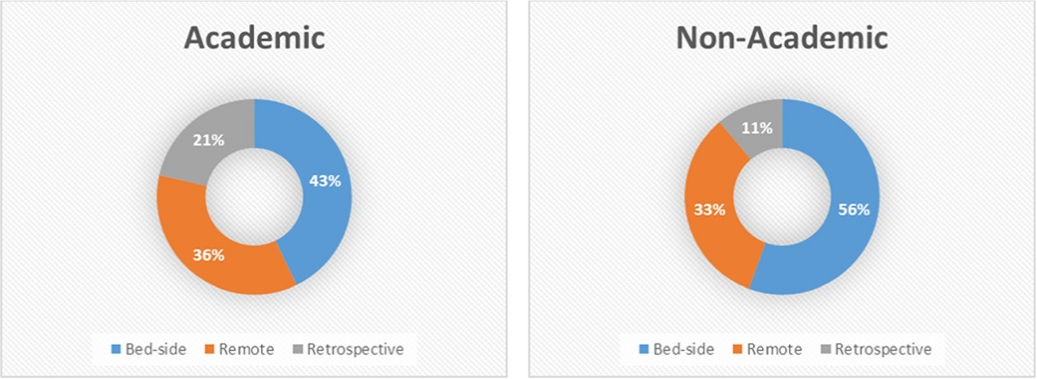
**Preferences for audit.**

The response (see Table 2) from respondents at academic hospitals is diverse and suggests audits are performed but still as a pilot in only a few wards. As Figure 1 depicts, there is no clear preference for bedside or remote consults as form of audit, but retrospective feedback seems less preferred.

At non-academic hospitals audits do not yet take place but are in either in development or considered necessary. Compared to academic hospitals the respondents of non-academic hospitals have a slightly stronger preference for bedside audits over remote audits via phone or email as can be seen in Figure 1. All respondents stated audit-and-feedback was the preferred strategy for ASP.

### Education

**Figure 2:**
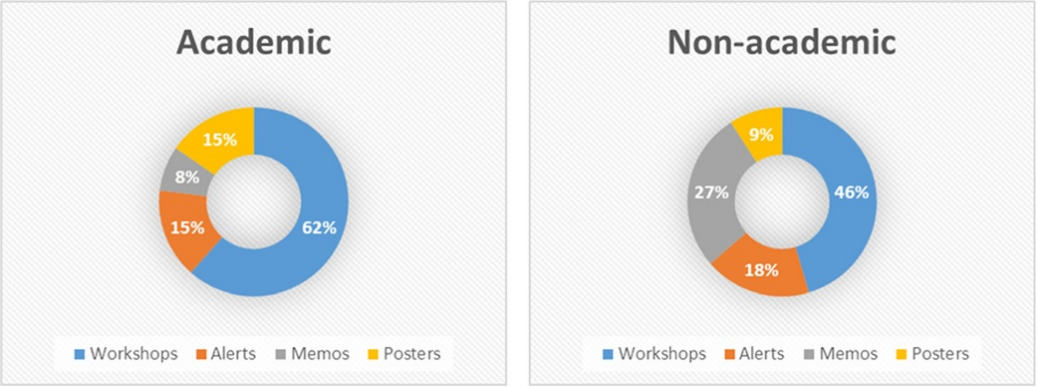
**Preferences for education.**

Educational activities are needed to disseminate the goals and interventions of ASP among prescribers and other possible stakeholders for ASP. At academic hospitals, educational activities implemented or in development relatively equally, suggesting it this is something that is being piloted and still taking shape. Non-academic hospitals have progressed less with implementing education and hence educational activities are still contemplated or in development. Respondents of both types of hospitals have a strong preference for workshops as means of education (Figure 2).

### Information systems for ASP

Specific information systems (information technology or software) that can be used in ASP were present at academic hospitals, except for information systems to evaluate prescriptions and decision support tools, e.g. for information while performing audits.

At non-academic hospitals, the presence of information systems was more divided than at their academic peers and not generalizable, but information systems for ASP are mostly in development Table 5.

**Table 5:** **Status of information systems for ASP in academic and non-academic hospitals**

	Academic hospitals					Non-academic hospitals				
	Impl	In dev	Need	Unneed	N/A*	Impl	In dev	Need	Unneed	N/A*
Electronic health records	5 (63%)	2 (25%)	1 (13%)	-	-	9 (82%)	2 (18%)	-	-	-
Digital laboratory data	6 (75%)	1 (13%)	1 (13%)	-	-	6 (55%)	1 (9%)	3 (27%)	-	1 (9%)
Digital antibiotic use data	7 (88%)	-	-	-	1 (13%)	4 (36%)	3 (27%)	2 (18%)	-	2 (18%)
Digital precribing	8 (100%)	-	-	-	-	7 (64%)	3 (27%)	1 (9%)	-	-
Evaluation of prescription	2 (25%)	4 (50%)	2 (25%)	-	-	1 (9%)	2 (18%)	5 (45%)	-	3 (27%)
Decision support systems	1 (13%)	2 (25%)	3 (38%)	-	2 (25%)	1 (9%)	4 (36%)	4 (36%)	1 (9%)	1 (9%)
Surveillance	6 (75%)	1 (13%)		-	1 (13%)	3 (27%)	4 (36%)	4 (36%)	-	-

*) Impl: implemented; in dev: in development; need: are needed; unneed: are unneeded; N/A: no answer or not applicable.

### Benchmarking

**Figure 3:**
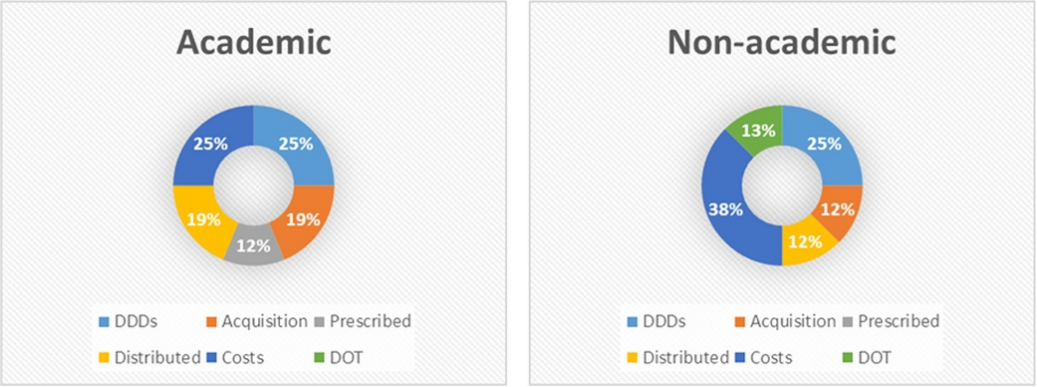
**Preferences for data sources for benchmarking.** *) DDDs: daily defined doses; DOT: days of therapy.

Benchmarking or monitoring antibiotic use related outcomes is necessary to assess changes caused by ASP. Such data can be compared internally (e.g. over time, between wards, between prescribers, etc.) and externally (e.g. between hospitals, nationally, internationally, etc.). Antibiotic teams at academic hospitals that benchmark or have it in development have access to common data sources for benchmarking. In non-academic hospitals benchmarking is less present and still in development, hence most data sources are not (yet) available either to the antibiotic team. Figure 3 gives an overview of the available (or in case of non-academic hospitals, in development) data sources. The figure suggests that daily defined doses and antibiotic costs are relatively the most used data types for benchmarking and monitoring antibiotic use.

### Restriction

Restriction of antibiotics means prescription of some or all antibiotics requires validation by a member of the antibiotic team. This intervention is not commonly present in academic hospitals, but stated as being in development. Current and future application of restrictive measures is more common in non-academic hospitals. None of the respondents indicated that restriction and (pre)-approval were identified as key strategies for ASP.

### Academic detailing

Academic detailing means that prescribers are influenced with structural evidence-based feedback, where the academic evidence plays an important role in the feedback as a form of education. This is hardly used in both types of hospitals but respondents stated this intervention is in either development or needed in the longer term. Currently there can be some ad-hoc academic explanation in feedback, but ‘academic detailing’ as a structural intervention that structurally provides prescribers with evidence-based feedback, is not seen as an important, urgent intervention according to our respondents.

### Automatic stop-order, pre-authorisation, automatic substitution and antibiotic cycling

Respondents of non-academic hospitals stated that these four interventions are all in development or needed. Academic hospitals appear to be only interested in developing automatic stop-orders or pre-authorisation as automatic substitution and antibiotic cycling were often stated as being unnecessary.

### Importance of ASP interventions

**Figure 4:**
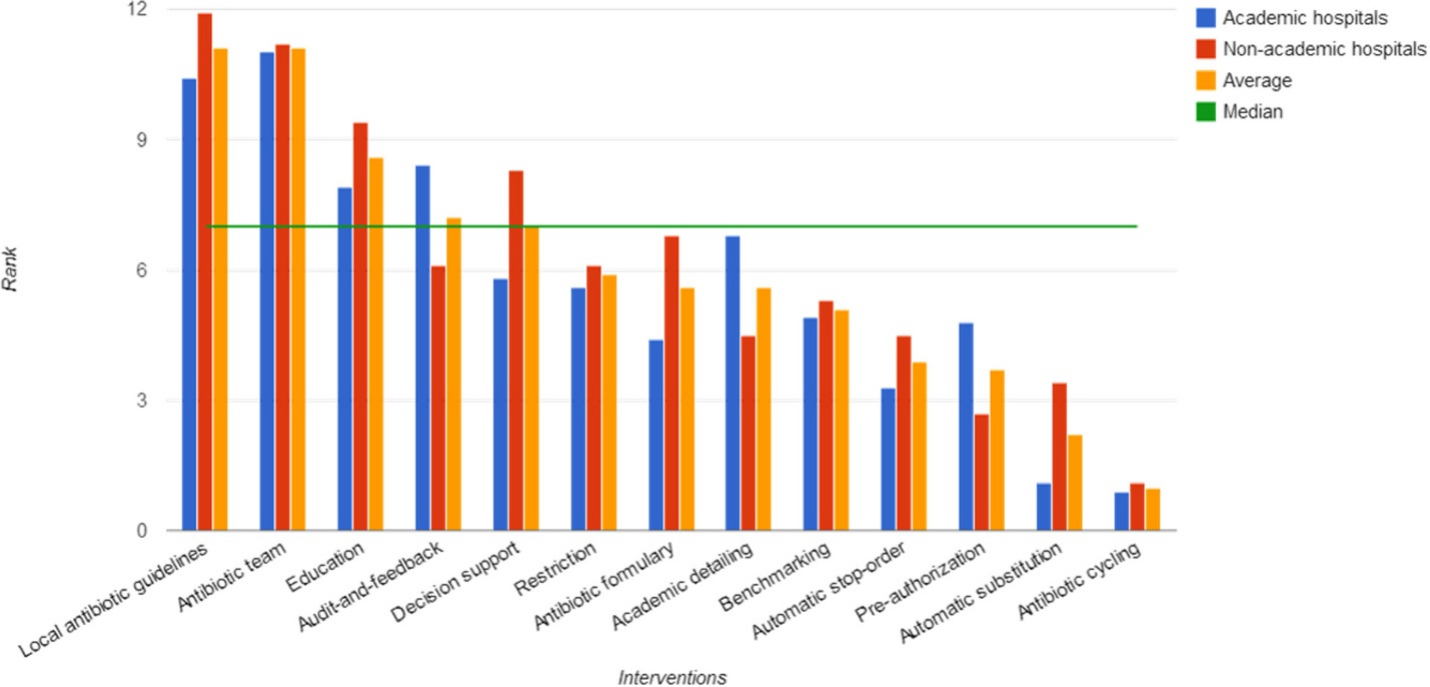
**Ranking of relative importance of ASP interventions.**

Respondents were asked in the questionnaire to rank ASP interventions. The list contained thirteen ASP interventions respondents could rank in order of importance to their ASP with a 1 to 13 score. Final scores in Figure 4 were averaged per type of hospital and overall average. The median of 7 was plotted to emphasize the perceived relative importance of interventions.

Local antibiotic guidelines and an antibiotic team are scored as the two currently most important interventions for implementing ASP in both types of hospitals. Education and audit-and-feedback scored relatively high as well, and in combination with earlier data in Table 2, they can be seen as part of a primary ASP bundle. Decision support systems receive more attention in non-academic hospitals than in academic hospitals. A possible explanation for this is the higher preference for bedside audits in non-academic hospitals (see Table 2) since for audits such decision support systems are particularly helpful. Less direct attention is needed for restrictive measures, an antibiotic formulary, academic detailing and benchmarking. We think an explanation for the low score for antibiotic formularies is that most Dutch hospitals already have such formularies in place and as such they are not directly seen as a specific, important intervention of ASP. The remaining interventions, automatic stop-order, pre-authorisation, automatic substitution, and antibiotic cycling score relative low on importance and it is questionable whether these are interesting interventions for ASP.

## Discussion

Both academic hospitals and non-academic hospitals in the Dutch border region are busy with antibiotic stewardship initiatives. When assessing local implementations of ASPs, a clear difference in academic and non-academic hospitals can be seen. Generally, academic hospitals are experimenting with comprehensive programs of recommended interventions in pilot ASPs. Non-academic hospitals have implemented interventions that are required by national guidelines and are expanding the program with other important interventions for a comprehensive ASP. Obviously the academic nature and larger size of academic hospitals support a more experimental, comprehensive approach, whereas non-academic hospitals seem to take a more practical approach by focusing on easily obtained gains first. Some existing processes can be reused as many activities that are now under the ASP umbrella are not necessarily new. For example, many hospitals have had remote consults with microbiologists in place for years. Also, this difference between academic and non-academic hospitals is strengthened by the fact that non-academic hospitals seem to have more difficulties in securing adequate resources for ASP.

When we combine maturity modelling with the findings of the current progress in ASP in hospitals included in our questionnaire, a number of conclusions can be drawn:

- In the state before ASP really starts, the null or m_0_ state of a maturity model, most Dutch hospitals already have a comprehensive antibiotic formulary and at least guidelines for treatment of infections, antibiotic therapy and prophylaxis. This is helpful as it can be a head start for implementing ASPs. In many other countries such an antibiotic formulary did not yet exist and is usually an important first ASP activity of the antibiotic team (van Limburg M, Köck R, Karreman J, Sinha B, de Jong N, Wentzel J, Friedrich A, Hendrix R, van Gemert-Pijnen J, “Towards an Implementation Strategy for Antibiotic/Antimicrobial Stewardship: A Systematic Review”, Under review).
- In the first stage of maturity, the initial phase, processes are ad-hoc and unorganised [11]. We found that hospitals are triggered by the SWAB vision document [9] and data from the questionnaire suggests that stakeholders are busy with a primary bundle of interventions that constitute an ASP. An antibiotic team, adequate local antibiotic guidelines for ASP, educational activities and an audit-and-feedback intervention receive early attention and seem to be the first interventions to be implemented when hospitals start with ASP. However, it seems each intervention is implemented quite differently, according to local contexts and readily available means. In other words, interventions are implemented with a slight variation between them. For example, an intervention that seems rather straight-forward is an antibiotic team, however, the composition of an antibiotic team is already quite different in each hospital, depending on the available staff, and care focuses (children, trauma, etc.) in that hospital.
- The second stage of maturity, a managed state, is what regulatory documents aim for. There is a risk that the current proliferation of local ASPs and local variations between interventions in hospitals are unstandardized and will therefore be difficult to regulate, compare and manage. For example, how does a team with an ID physician relate to a team without? Timely guidelines that help standardisation are necessary. From a regulatory perspective, two scenarios are possible: a) allow proliferation and wait until a dominant design emerges – assuming that over time current ASPs will evolve into comparable programs or b) interact with hospitals and understand the local differences and anticipate with the regulations.
- Further stages of maturity, which is a measured and self-optimising state, is difficult to achieve for ASPs in their current state. Standardisation in processes and measurements are necessary to evaluate ASPs both internally and externally. We found that data collection for benchmarking can be done; however, as ASPs are currently novel and diverse between hospitals, this would be like comparing apples and oranges. Also, although there is evidence that ASPs show positive effects on antibiotic use and antibiotic resistance [14, 15], there is little or no standardisation in how effectiveness of ASPs is measured in terms of standardised outcomes or even methodologies [16, 17]. That is also causing difficulties to compare (different) programs.

The assessment of the implementation of ASP can be expanded further by understanding the rationale and considerations local experts have based on expert-based recommendations in available regulatory documents. Patel et al. concluded that little guidance is offered on the practical aspects of implementing ASPs and that non-academic hospitals in the US need to overcome implementation issues by accounting for unique characteristics of their institutions [18]. Understanding these issues combined with these unique characteristics can eventually be used for parameters to customise local implementations of expert-based recommendations.

Earlier maturity assessments identified a set of structure indicators or characteristics that need to be present [13, 19]. Prudence should be shown to assess the maturity of ASP implementation when using these structure indicators, as local experts may have valid reasons to deviate from expert recommendations. For example, a non-academic hospital may not have infectious disease physicians but a clinical microbiologist performing similar tasks. If available maturity assessments are strictly observed, this example hospital is missing an important structure indicator, while in practice the ASP was implemented differently from the ideal program as proposed and assessed by experts. Therefore, tallying whether expert-recommended interventions are present is not enough to capture the progress and maturity of ASP implementations.

By applying convenience sampling, we contacted respondents indirectly via infection control experts in the Dutch eastern border region and we depended on their willingness and involvement with ASP to find and persuade colleagues to partake. Due to the novelty of ASP, some experts declined participation as they claimed they were not sufficiently progressed with ASP initiatives yet. As a result, this study assessed only nineteen stakeholders from nine hospitals in the Dutch border region. Conclusions of this study do not reflect the entirety of Dutch hospitals. However, this was not the primary goal of the study, as we wanted to assess the implementation process of early ASP initiatives, for which the sample size sufficed. Another limitation was that by reducing the number of questions, not every intervention was assessed in high detail. We also omitted questions regarding microbiological diagnostics, infrastructure or tests and policy forming to keep the questionnaire at an acceptable length.

To keep the momentum going of ASP implementations and to improve the chances of success of these implementations, future research is necessary to obtain further insights into the rationale and issues that local experts consider during local implementations. What considerations did they have and why? At academic hospitals it would be interesting to assess what was learned with experimentation in pilot implementations. How did they arrive at the interventions as used in pilots and how will these pilots be scaled up? Case studies of these pilots can lead to concrete examples of potential and different implementations that can be used for advising other hospitals various configuration possibilities of ASPs. At non-academic hospitals, future research will be more focussed on practical issues in implementing ASP. What do local experts in non-academic hospitals consider in practice when implementing ASP? What are easily obtainable gains? What are barriers?

These different case studies, potential issues and rationale behind characteristics between interventions can be used as parameters that influence the configuration of to-be implemented ASPs. By inventorying all these parameters, local experts can be supported in customising and implementing an ASP that fits their hospital. Eventually, these parameters could be used to synthesise an implementation maturity toolkit. We plan to design a decision aid for experts in academic and non-academic hospitals that will generate a customised implementation advice for ASP fit for their local conditions.

## Conclusion

Advising an ASP implementation is not straightforward. Experts who are tasked to introduce ASPs in their hospitals use expert-driven guidelines but need to transpose these guidelines to locally implemented interventions. This transposition leaves leeway for experimentation and considerations and leads to local differences in implemented intervention and ASPs. Progress can be made by assisting local experts with implementing ASPs in their hospitals. This assistance, however, needs to take into account that local conditions need to be translated into practical implementation advice and that a ‘one-ASP-for-all’ advice does not meet the needs of local experts. A bottom-up assessment with local experts can find parameters that influence local implementations of ASPs. These parameters can be used as input for a decision aid that generates a customised advice for a local implementation of an ASP.

## Electronic supplementary material

Additional file 1: **English translation of online questionnaire “Maturity assessment of antibiotic stewardship programs”.** (DOC 77 KB)
