# Supplementary material for: Evaluation of early implementations of antibiotic stewardship program initiatives in nine Dutch hospitals
Source: Antimicrob Resist Infect Control. 2014 Oct 23;3:33. doi: 10.1186/2047-2994-3-33 (PMC4228167; doi:10.1186/2047-2994-3-33)
Supplement: Supplementary file 1 — Additional file 1: English translation of online questionnaire “Maturity assessment of antibiotic stewardship programs”. (DOC 77 KB) [file 13756_2014_598_MOESM1_ESM.doc]

**Additional file 1: English translation of online** questionnaire “Maturity assessment of antibiotic stewardship programs”

| **Introduction** | | |
| --- | --- | --- |
| 1 | What is your profession? | Clinical pharmacist|clinical microbiologist|”infectioloog”. |
| 2 | Are there initiatives for antibiotic stewardship in your hospital? | Yes|in dev|no|N/A |
| 3 | Since what year? | XXXX |
| Topic #1: **ASP initiatives** | | |
| 4 | Pick an answer on the following statements:   - There is management mandate for ASP - There is a budget available for ASP - This budget is sufficient - Tasks are formally documented for ASP - There are FTEs available for ASP - These FTEs are sufficient - ASP can contribute substantially to infection prevention in my hospital | Strongly agree|agree|indifferent|disagree|strongly disagree|N/A|unknown |
| Topic #2: **Antibiotic team** | | |
| 5 | Does your hospital have an antibiotic team | Yes|in dev|no|N/A |
| 6 | How often does this team assemble? | Daily|weekly|monthly|semiyearly|yearly|N/A |
| 7 | Who are part of this antibiotic team?   - Clinical microbiologist - “Infectioloog”/ID physician - Clinical pharmacist - Management representative - Internist - Nurse - Antibiotic committee representative - Ward/supervising physician - (Prescribing) physician - Epidemiologist - Hygienist - IT specialist | In our team|Should be in a team|Should not be in a team|N/A |
| 8 | Are any disciplines missing in this list? | Open answer |
| 9 | A-teams play a crucial role in ASP implementations | Strongly agree|agree|indifferent|disagree|strongly disagree|N/A|unknown |
| Topic #3: **(Local) antibiotic guidelines** | | |
| 10 | Your hospital has guidelines for:   - Diagnosis of infections - Treatment of infections - Duration of treatment of infections - Antibiotic therapy - IV-PO switches - Streamlining/de-escalation of antibiotics - Antibiotic cycling - Peri-operative prophylaxis | Implemented|In development|Are needed|Are not needed|N/A |
| 11 | Antibiotic guidelines play a crucial role in ASP implementations | Strongly agree|agree|indifferent|disagree|strongly disagree|N/A|unknown |
| Topic #4: **Antibiotic formulary** | | |
| 12 | Does your hospital have an antibiotic formulary? | Yes|in dev|no|N/A |
| 13 | Is this formulary based on local resistance patterns? | Yes|no|N/A |
| 14 | How often is checked if prescription reflects antibiotic formulary? | Always|often|occassionally|rarely|never|N/A |
| 15 | Antibiotic formulary plays a crucial role in ASP implementations | Strongly agree|agree|indifferent|disagree|strongly disagree|N/A|unknown |
| Topic #5: **Audit-and-feedback** | | |
| 16 | Does your hospital perform audits? | Yes|in dev|no|N/A  Other -> open answer to “Please elaborate how your hospital reviews antibiotic therapy” |
| 17 | Who is performing these audits? | Clinical pharmacist|clinical microbiologist|”infectioloog”|other… |
| 18 | Which forms do these audits or should these audits have? | Bedside consult|remote consult(email/phone)|retrospective feedback(not during therapy)|other…|N/A |
| 19 | The person doing the audit takes action when a prescription is not conform guidelines | Strongly agree|agree|indifferent|disagree|strongly disagree|N/A|unknown  Other -> open answer to “Please elaborate how the audit process is or should be” |
| 20 | Audits play a crucial role in ASP implementations | Strongly agree|agree|indifferent|disagree|strongly disagree|N/A|unknown |
| Topic #6: **Education** | | |
| 21 | Are there educational activities in your hospital for ASP? | Yes|in dev|no|N/A |
| 22 | Which forms of education? | Workshops|lectures|posters|memos|alerts|other… |
| 23 | Current educational activities are sufficient to improve prescription behavior | Strongly agree|agree|indifferent|disagree|strongly disagree|N/A|unknown |
| 24 | Education plays a crucial role in ASP implementations | Strongly agree|agree|indifferent|disagree|strongly disagree|N/A|unknown |
| Topic #7: **Information systems** | | |
| 25 | Are there IT/software systems dedicated to ASP available in your hospital? | Yes|in dev|no|N/A |
| 26 | Please provide the current status of these IT/software systems in your hospital   - Electronic Health Record - Decision support tools - Tracking antibiotic use - Surveillance - Sharing laboratory results - Digital prescribing - Evaluation of prescriptions - Digital patient file/”status” | Implemented|In development|Needed|Not needed|N/A |
| 27 | IT play a crucial role in ASP implementations | Strongly agree|agree|indifferent|disagree|strongly disagree|N/A|unknown |
| Topic #8: **Benchmarking** | | |
| 28 | Does your hospital update resistance figures every year? | Yes|No, but we should|No, not necessary|N/A |
| 29 | Does your hospital benchmark antibiotic usage periodically? | Yes|No, but we should|No, not necessary|N/A |
| 30 | How often? | Daily|weekly|monthly|semiyearly|yearly|N/A |
| 31 | Benchmarked on which level? | Ward|hospital|N/A|other… |
| 32 | This data is used for trend analyses | Yes|No, but we should|No, not necessary|N/A |
| 33 | This data is presented to prescribing physicians | Yes|No, but we should|No, not necessary|N/A |
| 34 | Please select in which measures this data is available. | DDDs|PDDs|DOT|acquisition quantities|distributed quanitities|antibiotic costs|other… |
| Topic #9: **Implementation of ASP** | | |
| 35 | On which level is ASP is implemented in your hospital? | Entire hospital|most wards|few wards|one ward|nowhere|N/A|unknown  In case of 1 or more wards open anwer to “Please specify which wards” |
| 36 | ASP should be implemented on which level? | Entire hospital|most wards|few wards|one ward|nowhere|N/A|unknown |
| 37 | The implementation of an ASP should be detailed on which level? | Hospital wide|per ward|Combination of both|N/A |
| 38 | Does your hospital collaborate with other hospitals in terms of ASP? | Yes|no|N/A |
| 39 | Please rank the following ASP interventions in order of importance for your hospital   - Antibiotic team - Academic detailing (evidence-based education) - Pre-authorization of antibiotics - Audit-feedback - Restriction on (reserve) antibiotics - (Automatic) stop-order - Antibiotic cycling - (Automatic) substitution - Antibiotic formulary - Education - Guidelines - Decision support tools - Benchmarking | [RANKING 1-13] |
| 40 | Please provide the current status of these ASP interventions in your hospital   - Antibiotic team - Academic detailing (evidence-based education) - Pre-authorization of antibiotics - Audit-feedback - Restriction on (reserve) antibiotics - (Automatic) stop-order - Antibiotic cycling - (Automatic) substitution - Antibiotic formulary - Education - Guidelines - Decision support tools - Benchmarking | Implemented|In development|Needed|Not needed|N/A |
| 41 | Are interventions missing in the list we provided in the above two questions? | Open answer |
| Topic #10: **Important outcomes to assess ASP** | | |
| 42 | Please rank the following ASP outcomes in order of importance for your hospital   - Antibiotic use - Length-of-stay - Prevalence rates - Cure/eradication rates - Days of therapy - Mortality - Morbidity - Resistance rates - Compliance rates - Costs - Re-admissions - Number of errors - Time spent per patient | [RANKING 1-13] |
| 43 | Are outcomes missing in the list we provided in the above question? | Open answer |
| **Questions for questionnaire administration** | | |
| 44 | What is the name of your hospital | Open answer |
| 45 | Do you want feedback of the results of this questionnaire? | Yes|no |
| 46 | Can we contact you for further research? | Yes|no  Yes->Open answer to “Please provide us your email address” |
| 47 | Any questions, advice or other remarks you want to share with us? | Open answer |
